# Preparation and evaluation of functional cocoa‐free spread alternatives from different sources

**DOI:** 10.1002/fsn3.4095

**Published:** 2024-04-12

**Authors:** Sayed Saad Smuda, Amera T. Mohammed, Efstathia Tsakali, Jan F. M. Van Impe, Asmaa M. Marie

**Affiliations:** ^1^ Food Science Department, Faculty of Agriculture Cairo University Giza Egypt; ^2^ Department of Crops Technology Research Food Technology Research Institute, Agricultural Research Center Giza Egypt; ^3^ Department of Food Science & Technology University of West Attica Egaleo Greece; ^4^ BioTeC+‐ Biochemical Process and Control, Department of Chemical Engineering KU Leuven Technology Campus Gent Gent Belgium

**Keywords:** cocoa alternatives, cocoa‐free spread, sensory attributes physicochemical characteristic, storage stability

## Abstract

Spread products have an important market share as they have high nutritional value and they are increasingly consumed, especially by children as a source of energy. The purpose of this work was to evaluate the potential use of powdered chickpea, black rice, carob, doum, date seeds, and beetroot to produce novel functional spreadable products as cocoa‐free alternatives. Additionally, to avoid the side effects of cocoa‐based products and to assess the cocoa replacement effects on the sensory properties, chemical composition, texture analysis, viscosity, antioxidant, peroxide stability, and microbial quality during storage periods were compared to the ones of cocoa spread. Sensory evaluation revealed that most formulated spreads were accepted as chocolate spread alternatives since there was no significant difference in overall acceptability among cocoa, chickpea, black rice, carob, and doum, while date seeds and beetroot spreads were significantly less acceptable. A variation was observed in the proximate chemical analysis of the produced functional spreads, as the alternative spreads had different characteristics to each other in their physicochemical, texture, and rheological properties. Results indicated a wide variation in the total phenolic content (TPC) of the different spread extracts. The highest amount of TPC was obtained for beetroot spread (455.84 mg GAE/100 g) followed by black rice spread (436.08 mg GAE/100 g). The obtained results indicated that the antioxidant activity of different spreads was significantly different (*p* < .05) while based on their microbiological analysis, they could have a shelf life of up to 9 months. According to the results, chickpea, carob, doum, black rice, date seeds, and beetroot powders could be used for the production of cocoa‐free alternatives as they were highly acceptable and they showed antioxidant and antimicrobial activity.

## Graphical abstract

1. Functional cocoa‐free spread was successfully prepared.
2. The potential of powdered hulled chickpea, black rice, carob, doum, date seeds, and beetroot as cocoa alternatives.
3. Formulation of novel functional/alternative spreads.
4. A variation in the proximate chemical analysis of the produced functional spreads was found.
5. The experimental functional cocoa‐free products had competitive nutritional compositions.
6. Microbial load of spread samples was within the acceptable limits for their shelf life of 9 months.

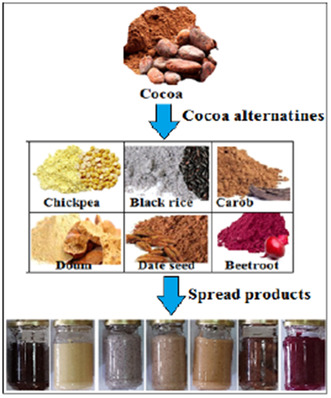

## 1 INTRODUCTION

Cocoa spread is an alternative product to traditional dry chocolate. It is a paste with sweet chocolate taste, consumed with bread, pancakes, and biscuits. It does not solidify at room temperature and is not recommended to be stored in the fridge. Also, it should have a creamy texture, light density without oil separation, and a shelf life of 6–12 months. Chocolate spreads usually contain cocoa powder, palm oil, sugar, and additional flavor (Said et al., 2019).

Cocoa (*Theobroma cacao* L.) has high dietary fiber content as well as phytochemical compounds and it is the base material used in the manufacture of chocolate. As it contains high amount of fat, the produced spreads have high energy value, and therefore, the extended consumption of cocoa products could lead to obesity (Caliskan et al., 2022). In addition, it contains caffeine that stimulates the central nervous system (Pandey et al., 2020). Consuming large amounts of cocoa may cause caffeine‐related side effects such as jitteriness, rapid heartbeat, insomnia, and increased urination. Cocoa contains allergenic substances that may cause allergic skin reactions and trigger migraines. Furthermore, it is considered an unhealthy product since a large amount of sugar is usually added during chocolate manufacturing due to the bitter taste that characterizes pure cocoa (García‐Díez et al., 2022).

Carob (*Ceratonia siliqua* L.) powder has high nutritional value, low‐fat, nutty flavor, is free of caffeine, and is often used as a natural sweetener and an alternative to cocoa (Morais & Rodrigues, 2018). It has high dietary fiber and low energy value, so it can be used as a substitute for cocoa powder‐based products to help remedy obesity (Caliskan et al., 2022; Issaoui et al., 2021). Thus, it is considered beneficial for human health and can be used to prepare healthy drinks and dessert products (Higazy et al., 2018; Ibrahim et al., 2020). Carob powder is often used for functional food formulations and is a cheap source of natural polyphenols for protection against many cardiovascular and neurological diseases (Ibrahim et al., 2015).

Black rice is a highly nutritional food due to its high total phenolic compounds, anthocyanins, and antioxidants. These compounds are biologically active, have scavenging activity against free radicals, and assist in good health preservation. Also, it contains vitamins E and B, carbohydrates, fats, proteins, dietary fiber, minerals, lysine, tryptophan, and other essential amino acids. Furthermore, it strengthens the digestive system, stabilizes high blood pressure, reduces allergies, controls diabetes, and it can assist against problems such as heart diseases, Alzheimer's disease, and atherosclerosis. Therefore, black rice has many applications due to its antioxidant properties and attractive purple color, which makes it a valuable component in the food industry (Khalil & Elkot, 2020; Panda et al., 2022).

Doum fruit (*Hyphaene thebaica*) reduces the serum's total cholesterol, total glyceride, and LDL as well as increases the HDL level due to its content of phytochemical compounds, which shows good antioxidant activity and scavenges free radicals (Abdullahi et al., 2022). Moreover, the presence of flavonoids and phenols in doum extract may be responsible for the improvement of blood glucose levels, blood liver enzymes, and lipid profiles (blood triglycerides, total fats, and cholesterol) (Abd‐Fatteh & Hassan, 2020). Food products fortified with doum can be beneficial for people who suffer from obesity and hyperlipidemia (Shehata, 2020).

Beetroot (*Beta vulgaris* L.) is classified as an excellent source of phytochemicals, which have anti‐inflammatory, antianemic, antioxidant, anticancer, and antibacterial activities as well as they can be beneficial in intestinal peristalsis, improving lipid metabolism, preventing Alzheimer's disease, and decreasing of aging (Babarykin et al., 2019; El‐Mesallamy et al., 2020). Beetroot is a superior source of iron which can assist in malnutrition and anemia; thus, it can be used in functional food production (Abdo et al., 2020). Moreover, it is a good source of protein, carbohydrates, dietary fiber, minerals, vitamin C, betalain, carotenoids, polyphenols, and flavonoids (Kale et al., 2018). Red‐colored beetroots are the most commonly used, but their ability to change color by heating allows their use in ice cream, puddings, and other sweets while they have no known allergy side effects (Neha et al., 2018).

Chickpea is an important leguminous, rich in protein (about 18%–22%), dietary fiber, several bioactive components, iron, calcium, and zinc. Subsequently, their addition is recommended to gluten‐free and casein‐free food products such as milk drinks and snacks intended for autistic patients (Ibrahim, 2022). They have several health benefits like anti‐diabetic, anti‐inflammatory, and hypo‐cholesterolemic activity while they are related to the prevention of cardiovascular diseases and they can be beneficial for people suffering from protein‐energy malnutrition and patients suffering from gluten intolerance (Kaur & Prasad, 2021). Chickpeas have shown significantly high bioavailability of iron and play an important role in the supply of protein and mineral micronutrients especially iron to people who depend on plant‐based diets for total calories (Jahan et al., 2019).

The seeds of date palm (*Phoenix dactylifera* L.) contain proteins, dietary fiber, fatty acids, proteins, and other phytochemicals (Gul et al., 2022). Therefore, they can be used effectively as a natural source of antioxidants and antibacterial for the development of different products such as functional foods, healthy innovative foods, and dietary supplements (Abuelgassim et al., 2020; Radfar et al., 2019).

The aim of the current study was to utilize different alternative sources such as chickpea, black rice, carob, doum, date seeds, and beetroot for the production of products similar to spreadable chocolate in terms of high nutritional value and high consumer acceptability without the allergic side effects. The produced spreads were further evaluated as per their nutritional properties, texture, and sensory properties as well as the storage stability.

## 2 MATERIALS AND METHODS

### 2.1 Materials

Cocoa, chickpea split, black rice, carob, doum, date seeds, beetroot, and other ingredients such as sugar, sunflower oil, vanillin, skim milk, and skim milk powder were obtained from the local market in Giza‐Egypt. Plate count agar (PCA) and mannitol salt agar were obtained from Merck (Darmstadt, Germany). All used chemicals were of analytical grade.

### 2.2 Methods

#### 2.2.1 Preparation of raw materials

The date seeds were dried in an air oven at 60 ± 5°C overnight and milled in a heavy‐duty grinder to obtain a powder. Beetroot tubers were washed with tap water, peeled, cut into slices, air dried, and then ground into powder. The other dry raw materials (chickpea split, roasted carob pods, doum, and black rice) were milled into a powder using an electric grinder (Moulinex®). All powders were sieved to obtain a fine powder to pass through a sieve (250 microns), then packed into polyethylene bags, and refrigerated until further use.

#### 2.2.2 Preparation of spread formulas

Chocolate spread (control) was prepared by mixing cocoa powder, sugar, skimmed milk powder, and vanillin in skimmed liquid milk before sunflower oil was added. Spreads were produced at maximum speed of electric mixer (Moulinex®). Cocoa powder was replaced (at the rate of 100%) with the other cocoa substitutes (chickpea, carob, doum, black rice, date seeds, and beetroot); seven formulas were prepared as shown in Table 1. The prepared formula spreads were poured immediately into sterilized glass jars and stored under refrigeration for further analysis. Spread samples were stored for 9 months.

**TABLE 1:** Formula of the produced spread samples (g/100 g).

Ingredients	%
Cocoa or substitutes^a^	14.42
Sunflower oil	13.43
Sugar	14.42
Skimmed milk powder	28.86
Skimmed liquid milk	28.86
Vanillin	0.01

^a^ Cocoa substitutes are chickpea, black rice, carob, doum, date seeds, and beetroot.

#### 2.2.3 Physicochemical properties of spread formulas

##### Chemical analysis

Moisture, crude protein, ash, fat, and crude fiber contents were determined according to AOAC (2010) methodology. Total carbohydrates were estimated by the difference computationally.

The energy of spread formula was calculated by using the following equation (James, 1995):

$$\text{Energy}\;\left(\text{Kcal}/100\;g\right)=\left(\mathrm{Fat}\times 9\right)+\left(\text{Protein}\times 4\right)+\left(\text{Total carbohydrate}\times 4\right)$$

##### Color measurement

Color of the produced spread samples was measured according to the method proposed by McGurie (1992) using a hand‐held Chromameter (model CR‐400, Konica Minolta, Japan). The parameters measured were lightness (L*) values from 0 (for black) to 100 (for white); redness‐greenness values (a*) are negative for green color and positive for red color; and yellowness (b*) negative values (for blue) and positive values (for yellow). All spread samples were analyzed in triplicate.

#### 2.2.4 Phytochemical characteristics

Total phenolic content was determined using Folin–Ciocalteu reagent according to the method of Singh et al. (2002). It was expressed as milligrams of gallic acid equivalents per 100‐g dry weight (mg GAE/100 g). The total flavonoid content (as milligrams of catechin equivalent per 100‐g dry weight) was evaluated using the method of Eghdami and Sadeghi (2010). The free radical scavenging activity was determined using the 2.2‐diphenyl‐2‐picryl‐hydrazyl (DPPH) method according to Fischer et al. (2013). The scavenging activity was calculated using the following equation:

$$\text{DPPH radical scavenging activity}\;\left(\%\right)=\left[\left(A_{0}–B_{1}\right)/A_{0}\right]\times 100$$

where *A*_0_ and *B*_1_ are the absorbance (at 515 nm) of the control and the sample, respectively.

#### 2.2.5 Apparent viscosity

The packed spread formulas were placed in a 40°C water bath to reach the temperature at which viscosity measurement was taken. Brookfield viscometer DV‐III ultra was used to measure the spread viscosity (centipoises, cps) using a spindle at a shear rate of 50 rpm for 30 s. Viscosity for all sample formulas was measured according to the method of Tizazu et al. (2010).

#### 2.2.6 Textural profile analysis (TPA)

Texture measurements of the produced spread samples were carried out on a Universal Testing Machine (Cometech, B type, Taiwan) equipped with software, according to the method of Bourne (2003). Firmness (hardness, N), cohesiveness (%), and adhesiveness (mJ) were calculated using the obtained TPA graphic. Hardness was denoted as the maximum force required to obtain accurate probe deformation (N). Cohesiveness was defined as the strength of the internal bonds within the particles of the sample. Adhesiveness was expressed as the used force to withdraw the probe from the sample and the withdrawal time.

#### 2.2.7 Sensory evaluation

The sensory acceptability of appearance, taste, color, aroma, spreadability, and overall acceptability of the produced spreads were evaluated. Ten panelists (from the staff members of the Food Technology Research Institute, Agricultural Research Center, Giza, Egypt) were chosen on the basis that they regularly consumed chocolate spreads. Panelists were asked to evaluate the hedonic attributes using a 9‐point hedonic scale ranging 1 = dislike very much, 5 = like slightly, 9 = like very much (Said et al., 2019).

#### 2.2.8 Microbial analysis

The microbiological quality of spread formulas was evaluated according to the method of Amevor et al. (2018). Total plate count and fungal count (yeasts and mold) were tested by serial dilution followed by solidification in petri plates using total count media and malt yeast agar media, respectively. After solidification, both bacteria and fungus colony‐containing plates were incubated at 37°C for 48 h. Then, the formed colonies were counted and converted as the number of colony‐forming unit (CFU) per gram of fresh sample as well as after 3, 6, and 9 months. Psychrotrophs were determined in plate count agar (PCA) after 10 days of incubation at 7°C (Şahin & Özata, 2022).

#### 2.2.9 Determination of oxidative stability (hydroperoxide content)

Hydroperoxide content of the extracted fat was determined according to Tarakçi and Yildirim (2020). The hydroperoxide content was determined using a standard curve prepared with known concentrations of cumene hydroperoxide. Concentrations were expressed as mmol hydroperoxide/kg of fat.

#### 2.2.10 Feasibility study

The feasibility study of spread formulas was carried out according to the method of Zoani et al. (2018).

#### 2.2.11 Statistical analysis

The data obtained were statistically analyzed using one‐way analysis of variance (ANOVA) using Costat statistical software based on a probability of *p* < .05. Mean values and standard deviations were reported for the analytical data.

## 3 RESULTS AND DISCUSSION

### 3.1 Physicochemical properties of spread formulas

#### 3.1.1 Chemical analysis

The results of the chemical analysis are presented in Table 2. The moisture content values ranged from 22.19% to 24.30%. The black rice spread formula had the lowest moisture content, while the chickpea spread formula had significantly the highest. The chickpea spread formula also had the significantly highest protein content (18.03%), while the lowest was observed in the carob spread formula (12.49%).

**TABLE 2:** Proximate chemical analysis and nutritional quality of spread formulas.

Parameter	Cocoa (control)	Chickpea	Black rice	Carob	Doum	Date seeds	Beetroot
Chemical composition (%)							
Moisture	22.79± 0.24^d^	24.30 ± 0.32^a^	22.19 ± 0.22^e^	23.42 ± 0.12^b^	23.20 ± 0.19^b^	22.85 ± 0.87^c^	22.93 ± 0.49^c^
Protein	17.03 ± 0.12^b^	18.03 ± 0.31^a^	15.16 ± 0.13^c^	12.49 ± 0.20^e^	12.94 ± 0.30^d^	14.75± 0.08^c^	15.18 ± 0.18^c^
Ash	7.80 ± 0.02^a^	5.03 ± 0.05^c^	3.94 ± 0.01^g^	4.59 ± 0.03^e^	5.22 ± 0.03^b^	4.27 ± 0.04^f^	4.84 ± 0.03^d^
Fats	35.54 ± 0.12^b^	38.82 ± 0.24^a^	31.51 ± 0.26^c^	29.94 ± 0.30^d^	29.01 ± 0.27^d^	34.77 ± 0.16^b^	24.35 ± 0.29^e^
Crude fiber	0.16 ± 0.05^d^	0.21 ± 0.02^d^	0.40 ± 0.02^d^	1.24 ± 0.06^c^	4.21 ± 0.01^b^	4.90 ± 0.24^a^	1.07 ± 0.02^c^
TC	39.47 ± 0.32^e^	37.91 ± 0.15^f^	48.99 ± 0.21^c^	51.74 ± 0.17^b^	48.62 ± 0.18^c^	41.31 ± 0.12^d^	54.56 ± 0.22^a^
Color characteristics							
L*	39.06 ± 0.03^f^	69.11 ± 1.13^a^	55.06 ± 0.40^c^	58.12 ± 0.47^b^	58.97 ± 0.70^b^	46.89 ± 0.29^d^	40.69 ± 0.35^e^
a*	1.81 ± 0.02^e^	−2.31 ± 0.08^f^	1.63 ± 0.03^e^	2.49 ± 0.13^d^	5.23 ± 0.17^b^	3.18 ± 0.09^c^	7. 30 ± 0.55^a^
b*	1.27 ± 0.24^f^	17.18 ± 0.60^a^	1.85 ± 0.11^e^	9.27 ± 0.05^c^	15.86 ± 0.08^b^	4.07 ± 0.18^d^	1.38 ± 0.07^f^

*Note*: Protein, ash fat, and crude fiber are calculated on a dry basis, TC is total carbohydrates, L* = lightness (L* = 100 for lightness and L* = zero for darkness), a* = a chromaticity on a green (−) to red (+) and b* = a chromaticity on a blue (−) to yellow (+). All values are means of three replicates ± *SD*, numbers in the same row followed by different letters are significantly different (*p* < .05).

The ash content of the cocoa spread formula was significantly higher compared to the other spread formulas (7.80%) while the lowest was observed in the black rice spread formula (3.94%). Concerning the fat content, the beetroot spread formula had the lowest fat content (24.35%), while the chickpea spread had the highest (38.82%). Date seed spread formula had the highest amount of crude fiber followed by the doum formula (4.90% and 4.21%, respectively). The chickpea, black rice, and cocoa spread formulas were the lowest in crude fiber without significant differences between them. These results and differences were expected as different raw materials with various attributes were used to substitute cocoa.

#### 3.1.2 Color measurement

Color of the produced spread formulas was significantly affected by cocoa substitution. The degree of lightness (L*), redness (a*), and yellowness (b*) of spread formulas is given in Table 2. The highest L* and b* values (69.11 and 17.18, respectively) were recorded in the chickpea spread formula followed by doum and carob spread formulas, while the least L* value was found in cocoa spread (control) (39.06). On the other hand, the chickpea spread formula had the least redness (a*) value (−2.31) while the highest value was in the beetroot spread formula (7.30) which is associated with the red color (betalains) of the beetroot. The different colors of produced spread formulas may depend on the different concentrations of pigment content within each product (Walker et al., 2014).

**FIGURE 1:**
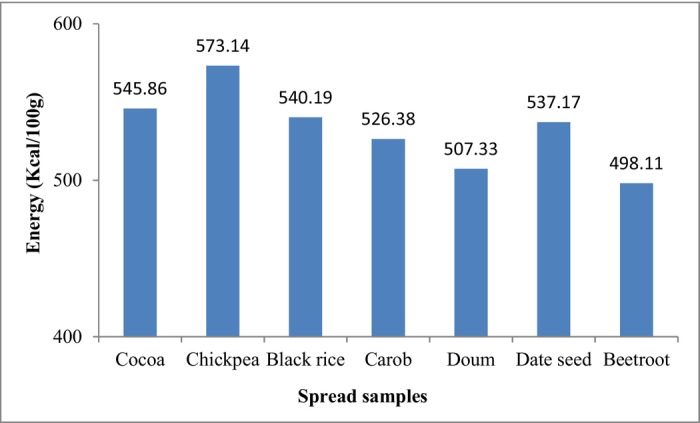
The energy of spread formulas.

The energy values of the spread formulas are shown in Figure 1. The highest energy value (573.14 Kcal/100 g) was found in the chickpea spread formula due to its high protein and fat content, while the beetroot spread formula had the lowest energy value (498.11 Kcal/100 g) due to its low‐fat content compared with the other spread formulas. A strong correlation of the energy values and the protein, fat, and carbohydrate contents of the samples occurs based on Equation (1).

#### 3.1.3 Nutritional values: Protein and energy

The dietary reference intake (DRI) of protein and energy per 100 g of spread formulas for males and females aged 9–13 years is shown in Table 3. Energy requirement (ER) is calculated as 2279 Kcal/day for males and 2071 Kcal/day for females. From the calculated data, chickpea spread formula seems to provide the highest DRI of protein and energy required for both males and females, while the carob spread formula offers the lowest DRI of protein (37%). Doum and beetroot spread formulas provide the lowest DRI values of energy for males and females.

**TABLE 3:** DRI of protein and energy required for males and females aged 9–13 years.

Spread formulas	DRI of protein for males and females	DRI of energy for males	DRI of energy for females
Cocoa	50	24	26
Chickpea	53	25	28
Black rice	45	24	26
Carob	37	23	25
Doum	38	22	24
Date seed	43	24	26
Beetroot	45	22	24
DRI**	Based on 34 g/day for males and females	Based on 2279 Kcal/day for males	Based on 2071 Kcal/day for females

*Note*: DRI** (2002–2005), DRI: (g/100 g spread).

### 3.2 Phytochemical compounds

#### 3.2.1 Total phenolic content, total flavonoid content, and antioxidant activity

The different spread formulas were analyzed for valuable phytochemicals such as total phenolic content (TPC) and total flavonoid content (TFC) and as per their antioxidant activity (AOA) (Table 4). Results indicated a wide variation in the TPC of the different spreads extracts. The highest amount of TPC was obtained for beetroot spread (455.84 mg GAE/100 g) followed by black rice spread (436.08 mg GAE/100 g). On the other hand, the lowest amount of TPC was reported in chickpea spread (198.88 mg GAE/100 g). Concerning total flavonoid content, the results revealed that chickpea spread contained the significantly highest value (155.28 mg CE/100 g) followed by beetroot spread (106.37 mg CE/100 g). Meanwhile, the cocoa spread contained the lowest value (62.96 mg CE/100 g). The evaluation of antioxidants is of great interest as they play an important role in inhibiting free radicals and delaying or inhibiting the oxidation of lipids (Nsimba et al., 2008). DPPH radical scavenging activity of the various spread formulas' extracts was determined. The obtained data showed that the antioxidant activities of the produced spread formulas were significantly different from each other (*p* < .05). The results showed that black rice spread had significantly the highest value of AOA compared to the other spread formulas. Although there was no significant difference in the AOA of carob, date seed, and beetroot formulas, still it was significantly higher than that of the control (cocoa).

**TABLE 4:** Total phenolic content, total flavonoid content, and antioxidant activities of spread samples.

Spread formulas	T. Phenolic	T. Flavonoids	Antioxidant activities
Cocoa	328.39^c^ ± 1.95	62.96^e^ ± 2.07	52.96^c^ ± 1.07
Chickpea	198.88^d^ ± 3.98	155.28^a^ ± 2.89	35.13^e^ ± 1.18
Black rice	436.08^a^ ± 2.66	70.39^d^ ± 3.33	68.17^a^ ± 2.10
Carob	428.99^ab^ ± 1.54	80.56^c^ ± 5.78	61.34^b^ ± 2.07
Doum	333.47^c^ ± 4.01	67. 32^de^ ± 5.48	44.52^d^ ± 1.03
Date seeds	394.18^b^ ± 1.68	81.07^c^ ± 3.72	62.15^b^ ± 2.09
Beetroot	455.84^a^ ± 3.45	106.37^b^ ± 1.65	63.13^b^ ± 1.43

*Note*: All values are means of three replicates ± *SD*, numbers in the same column followed by different letters are significantly different (*p* < .05).

### 3.3 Apparent viscosity

**FIGURE 2:**
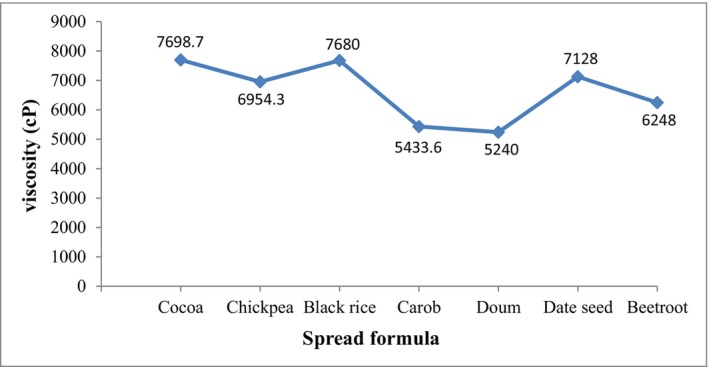
Apparent viscosity of spread formulas.

Rheological parameters of chocolate should be taken into consideration as they are affected by the composition and the processing treatment (Cozentino et al., 2022). Viscosity is a rheological property that measures the resistance of the fluid to deformation under stress and pouring, as it indicates the internal resistance of a fluid to flow and may be considered a measure of fluid friction (Akhtar et al., 2009; Geller & Goodrum, 2000). The viscosity (cP) of all spread formulas at room temperature is shown in Figure 2. The viscosity values indicated a noticeable variation, where the apparent viscosity was increased in the cocoa and black rice spreads (7698 and 7680 cP, respectively), which may be connected to the higher content of cellulose and pectin in cocoa and black rice (Mohidem et al., 2022; Redgwell & Hansen, 2000). Pectin is a hydrocolloid which is anionic in nature, used to gel and harden dairy products (Gulzar et al., 2015). The higher amount of starch (amylose and amylopectin) in the black rice sample may also have an effect (Yu et al., 2014). At the same time, the doum spread had the lowest viscosity values followed by carob spread, which can be explained by its higher concentration of endogenous sugars (Amagloh et al., 2013).

### 3.4 Texture profile analysis (TPA)

Texture analysis is considered very valuable for quality control and process optimization as well as for the development of new products with desirable properties (Aly & Seleem, 2015). The texture of spreads is one of the most effective factors for sensory acceptance and quality perception of food products. Hardness is described as the maximum force (N) required for the probe to penetrate the sample, higher hardness indicates lower spreadability (Bascuas et al., 2021). Texture profile analysis (TPA) of spread formulas is shown in Table 5. The doum spread formula showed the lowest hardness, while the cocoa spread formula had the highest hardness compared to all the other spread formulas. The variation of hardness of tested spread products can be explained by their different chemical composition (Glibowski et al., 2008).

**TABLE 5:** Texture profile analysis of spread samples.

Spread formulas	Hardness (N)	Adhesiveness (mJ)	Cohesiveness (%)
Cocoa	69.51 ± 0.02^a^	305.26 ± 0.03^b^	0.44 ± 0.01^f^
Chickpea	64.62 ± 0.02^b^	596.28 ± 0.02^a^	0.39 ± 0.02^e^
Black rice	30.52 ± 0.03^d^	168.27 ± 0.03^f^	0.48 ± 0.02^d^
Carob	41.62 ± 0.01^c^	215.09 ± 0.06^d^	0.45 ± 0.01^e^
Doum	23.91 ± 0.03^f^	111.44 ± 0.04^g^	0.52 ± 0.02^c^
Date seeds	27.90 ± 0.02^e^	231.20 ± 0.05^c^	0.56 ± 0.01^a^
Beetroot	27.92 ± 0.01^e^	187.60 ± 0.06^e^	0.54 ± 0.02^b^

*Note*: All values are means of three replicates ± *SD*, numbers in the same column followed by different letters are significantly different (*p* < .05).

Adhesiveness describes the necessary effect to overcome the attractive forces between the surface of the food sample and the surface that is in contact with the food sample (Dubost et al., 2003). Chickpea spread formula exhibited the highest adhesiveness among all spread samples which can be related to its increased protein and fat content (Murugkar, 2014). Meanwhile, the doum spread formula exhibited the significantly lowest.

Cohesiveness is the ability of elements of a material to stick together, which may also be defined as the strength of the internal bonds within the body of a product (Adhikari et al., 2001). Date seed spread formula showed significantly higher cohesiveness followed by the beetroot and the doum formulas (0.56%, 0.54%, and 0.52%, respectively). These results indicated that control (cocoa spread formula) was the significantly hardest formula compared with all prepared spread formulas. Adhesiveness and cohesiveness analysis revealed that the most adhesive and less cohesive products were with high fat content which comes to agreement with Glibowski et al. (2008). Furthermore, a rise in spreadability could also be attributed to fat content (Aydin & Özdemir, 2017).

### 3.5 Sensory evaluation

**FIGURE 3:**
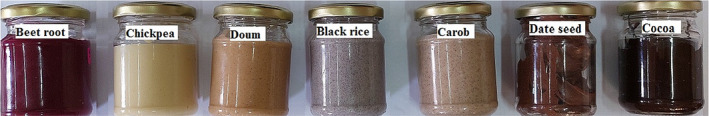
A photograph of the produced spread samples.

The results of the sensory evaluation of spread formulas are presented in Table 6 while their appearance is presented in Figure 3. No significant difference in the color acceptance of cocoa, chickpea, doum, date seed, and beetroot formulas was observed while they had a significantly higher score than carob and black rice formulas. Concerning the appearance, the data revealed that the cocoa and doum spread formulas recorded the highest appearance acceptability scores while there was no significant difference in the appearance of cocoa, doum, chickpea, carob, and beetroot spreads.

**TABLE 6:** Sensory evaluation of spread formula.

Sample	Color (9)	Appearance (9)	Spreadability (9)	Aroma (9)	Taste (9)	Melting quality (9)	OAA (9)
Cocoa	8.80 ± 0.63^a^	8.40 ± 1.26^a^	8.10 ± 0.90^c^	8.60 ± 0.96^a^	7.90 ± 0.90^a^	8.30 ± 1.22^ab^	8.25 ± 0.48^a^
Chickpea	8.02 ± 0.81^ab^	8.10 ± 0.87^ab^	8.20 ± 1.01^c^	8.05 ± 0.92^ab^	8.55 ± 0.49^a^	8.43 ± 0.47^a^	8.35 ± 0.62^a^
Black rice	7.12 ± 1.19^c^	7.00 ± 1.33^c^	8.45 ± 0.49^b^	7.65 ± 1.10^ab^	7.70 ± 1.41^ab^	7.40 ± 1.24^b^	7.60 ± 1.14^ab^
Carob	7.70 ± 0.91^bc^	7.85 ± 0.82^ab^	8.30 ± 1.03^b^	7.90 ± 1.10^ab^	8.55 ± 0.68^a^	8.45 ± 0.83^a^	8.25 ± 0.75^a^
Doum	8.35 ± 0.74^ab^	8.30 ± 0.48^a^	8.75 ± 0.42^a^	8.50 ± 0.81^a^	8.47 ± 0.44^a^	8.10 ± 0.84^ab^	8.35 ± 0.66^a^
Date seeds	8.05 ± 1.06^ab^	7.30 ± 1.22^bc^	8.68 ± 0.66^ab^	8.05 ± 1.06^ab^	7.40 ± 1.04^b^	7.35 ± 0.85^b^	7.40 ± 1.04^b^
Beetroot	8.00 ± 0.94^ab^	8.00 ± 0.81^ab^	8.65 ± 0.47^ab^	7.25 ± 1.08^b^	7.10 ± 0.77^b^	7.80 ± 1.03^ab^	7.25 ± 0.95^c^

*Note*: Data are presented as means of three replicates ± *SD* (a 9‐point hedonic scale). Values within the same column with different letters are significantly different (*p* < .05).

Abbreviation: OAA, overall acceptability.

Spreadability is the most important attribute affecting consumer acceptance of some products like jams, butter, and spread which is defined as the ease to apply a spread on a piece of bread or cracker. For this reason, the spread products should be easily spreadable to avoid tearing of the bread or crumbling of the crackers (Aydin & Özdemir, 2017), and the texture must be smooth, to immediately melt and not stuck on the mouth surface (Said et al., 2019).

The results indicated no significant differences in the spreadability of doum, date seeds, and beetroot formulas which were significantly the most spreadable formulas. This may be due to the lower fat and higher fiber content in these cocoa alternatives used as it has been reported that lower fat and higher fiber content aid in the spreadability of chocolate spreads (Amevor et al., 2018; Glibowski et al., 2008). Their increased spreadability may also be related to their lower hardness as indicated by TPA results (Table 3) which are in accordance with Said et al. (2019). The spread formula with beetroot had a significantly lower aroma score compared to the other formulas which showed no significant difference in aroma among them. Finally, no significant difference in taste and overall acceptability was recorded for cocoa, chickpea, black rice, carob, and doum formulas but they were more acceptable compared to date seed and beetroot formulas.

### 3.6 Microbiological quality (CFU/g) during storage

Bacterial count is considered a suitable parameter in order to predict the shelf life of a food product as microbial growth during the storage period and it is a critical factor for safety, quality, and organoleptic properties. Microbial analysis of all spread formulas was performed (total plate count, fungus, and psychrotrophs) on the production day as well as after 3, 6, and 9 months. The effect of the storage period on the microbial quality (CFU/g) of spread samples is shown in Table 7. The microbial load of the spread samples after the maximum storage period of 9 months was still acceptable. The bacterial count and fungal growth were lower than the recommended safety limit proposed by Codex Standard for Cocoa Products and Chocolate (CODEX STAN 87–1981, Rev. 1–2003, 2003). The maximum total plate count and fungal count after 9 months of storage were found in chickpea spread sample (45.5 × 10 CFU/g and 15 × 10, respectively). The variations of the microbial load were within the tolerable limits set by Codex Standard for Cocoa Products and Chocolate. On the other hand, carob spread recorded a high value of total Psychrotrophic bacteria (14 × 10 CFU/g). The microbiological stability may be related to antiviral, antibacterial, and antifungal properties of the used raw materials such as black rice, date seeds, beetroot, etc. Moreover, the high antioxidant activity of these materials which contain phenolic acids and flavonoids may contribute to the microbial stability and quality of the spread formulas.

**TABLE 7:** Microbiological quality (CFU/g) of spread samples during different storage periods.

Spread formulas	Storage period (months)											
	Total count				Mold and yeast				Psychrotrophs			
	0	3	6	9	0	3	6	9	0	3	6	9
Cocoa	1.5 × 10	6.5 × 10	30 × 10	39 × 10	ND	1 × 10	6 × 10	11 × 10	ND	2.5 × 10	9 × 10	13.5 × 10
Chickpea	2 × 10	8.5 × 10	43.5 × 10	45.5 × 10	ND	2 × 10	8 × 10	15 × 10	ND	3 × 10	10.5 × 10	12.5 × 10
Black rice	1.5 × 10	4.5 × 10	13 × 10	18.5 × 10	ND	ND	3 × 10	6 × 10	ND	1.5 × 10	7.5 × 10	8.5 × 10
Carob	4 × 10	9.5 × 10	28 × 10	36 × 10	ND	1 × 10	10 × 10	13 × 10	ND	3 × 10	10.5 × 10	14 × 10
Doum	1 × 10	8.5 × 10	31 × 10	37.5 × 10	ND	2 × 10	6 × 10	14 × 10	ND	2.5 × 10	9.5 × 10	12.5 × 10
Date seeds	3 × 10	6.5 × 10	16 × 10	24 × 10	ND	ND	4 × 10	8 × 10	ND	2 × 10	8.5 × 10	10.5 × 10
Beetroot	2 × 10	7.5 × 10	19 × 10	32 × 10	ND	ND	5 × 10	11 × 10	ND	2.5 × 10	9 × 10	13 × 10

The microbiological safety of chocolate spreads is secured by the good manufacturing practices adopted during their production, the high concentrations of sugars and fats, and the low water activity of the products and the proper maintenance of this parameter throughout the storage period (Zyzelewicz et al., 2010).

### 3.7 Lipid hydroperoxides (peroxide value)

**FIGURE 4:**
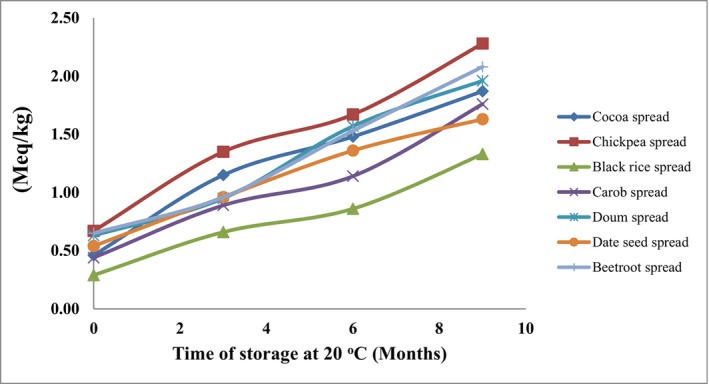
Lipid hydroperoxides of spread samples.

The primary oxidation of unsaturated lipids was measured by the hydroperoxide concentration (Figure 4). When stored at 20°C, the hydroperoxide peak for chickpea, beetroot, doum, cocoa, carob, date seeds, and black rice spreads was 2.28, 2.08, 1.96, 1.87, 1.76, 1.63, and 1.33 meq/kg, respectively, after 9 months of storage. Thereafter, the hydroperoxide decomposition rate was increased.

Hamid and Damit (2004) evaluated cocoa butter stability during storage at 15 and 70°C and observed that the increase in temperature anticipated the peroxide peak from 4 to 6 months. The peroxide value (PV) observed in the spread formulas during the shelf life study was lower than 5.0 meq O_2_/kg. This value can be considered similar when compared with PV of other fresh vegetable oils, such as coconut (4.9 meq O_2_/kg), soybean (2.4 meq O_2_/kg), or canola (5.0 milli equivalent O_2_/kg) (Chaiyasit et al., 2007). These results suggest a possible antioxidant effect of spreads supported by natural antioxidant compounds of the raw ingredients. A general rule is that PV should not be above 20 meq/kg fat to avoid rancidity flavor (Olusola, 2007). The peroxide value of fresh oil or fats is usually less than 10 meq/kg while it is usually above 20 meq/kg for rancid oils and fats (Eagan & Kir, 1981).

### 3.8 Feasibility study

Table 8 shows the feasibility study of the produced spread samples. All produced spread formulas showed lower production costs compared to the cocoa spread (control) which are directly affected by the lower price of the used cocoa substitutes. Date seed spread showed the lowest production cost (10.9), followed by beetroot spread (11.3).

**TABLE 8:** The feasibility study of spread formulas (100 g).

Ingredients	%	Cocoa spread	Chickpea spread	Carob spread	Doum spread	Black rice spread	Date seed spread	Beetroot spread
Cocoa or substitutes^a^	14.42	3.3	1.4	0.7	0.9	0.7	0.1	0.4
Sunflower oil	13.43	1.0	1.0	1.0	1.0	1.0	1.0	1.0
Sugar	14.42	0.4	0.4	0.4	0.4	0.4	0.4	0.4
Skimmed milk powder	28.86	7.0	7.0	7.0	7.0	7.0	7.0	7.0
Skimmed liquid milk	28.86	0.7	0.7	0.7	0.7	0.7	0.7	0.7
Vanillin	0.01	0.3	0.3	0.3	0.3	0.3	0.3	0.3
Energy 15%	–	1.9	1.6	1.5	1.5	1.5	1.4	1.5
Total cost/100 g	–	14.6	12.4	11.6	11.8	11.6	10.9	11.3

^a^ Cocoa substitutes are chickpea, carob, doum, black rice, date seeds, and beetroot.

## 4 CONCLUSION

Novel functional cocoa‐free spreads were developed using cocoa powder substitutes, with a long shelf life as well as good consumer acceptance. Chickpea, carob, doum, black rice, date seeds, and beetroot powders were used as cocoa replacers. In terms of overall acceptability and sensory quality, the chickpea, carob, doum, and black rice spread formulas were significantly more acceptable than date seed and beetroot spread formulas. A variation was observed in the proximate chemical analysis of the produced functional spreads. The chickpea spread with the significantly highest contents of moisture, protein, and fat exhibited the highest energy value while beetroot spread had the lowest value. All produced cocoa‐free spreads were significantly less hard compared to the control. Furthermore, all produced spreads were significantly less adhesive than the control except the chickpea spread which was more adhesive. The microbial load of the produced spread formulas was under the acceptable limits for a storage period of 9 months. Low peroxide values were also observed during the shelf life study which may be related to the antioxidant effect of natural antioxidant compounds of the raw ingredients used as cocoa substitutes.

## AUTHOR CONTRIBUTIONS

**Sayed Saad Smuda:** Conceptualization (equal); methodology (equal); resources (equal); validation (equal); visualization (equal); writing – original draft (equal); writing – review and editing (equal). **Amera T. Mohammed:** Investigation (equal); methodology (equal); validation (equal); writing – original draft (equal); writing – review and editing (equal). **Efstathia Tsakali:** Investigation (equal); validation (equal); visualization (equal); writing – review and editing (equal). **Jan F. M. Van Impe:** Investigation (equal); validation (equal); visualization (equal); writing – review and editing (equal). **Asmaa M. Marie:** Conceptualization (equal); investigation (equal); methodology (equal); resources (equal); validation (equal); visualization (equal); writing – original draft (equal); writing – review and editing (equal).

## FUNDING INFORMATION

This research was not funded nor received any specific grant.

## CONFLICT OF INTEREST STATEMENT

The authors do not have any conflict of interest to declare.

## Data Availability

Data are included within the article and data are not shared.

## References

[fsn34095-bib-0001] Abd‐Fatteh, N. , & Hassan, E. H. (2020). Effect of doum fruit (*Hyphaene thebaica*) extract on some biochemical parameters, enzyme activities and histopathological changes of pancreas in Alloxan induced diabetic rats. Food and Nutrition Sciences, 11(3), 207–219. 10.4236/fns.2020.113016

[fsn34095-bib-0002] Abdo, E. , El‐Sohaimy, S. , Shaltout, O. , Abdalla, A. , & Zeitoun, A. (2020). Nutritional evaluation of beetroots (*Beta vulgaris* L.) and its potential application in a functional beverage. Plants, 9, 1752–1769. 10.3390/plants9121752 33322047 PMC7764643

[fsn34095-bib-0003] Abdullahi, A. N. , Abdulmumin, Y. , Abdulmumin, T. M. , Sheshe, S. M. , Ismail, S. Y. , Murtalat, M. , Ibrahim, A. M. , Hassan, M. K. , Bichi, S. A. , Sarki, S. I. , & Abubakar, S. (2022). Hypolipidemic effect of aqueous fruit extract of doum palm (*Hyphaene thebaica*) in Wistar rat. Chemical and Pharmaceutical Research, 4(3), 1–4. 10.33425/2689-1050.1040

[fsn34095-bib-0004] Abuelgassim, A. O. , Eltayeb, M. A. , & Ataya, F. S. (2020). Palm date (*Phoenix dactylifera*) seeds: A rich source of antioxidant and antibacterial activities. Czech Journal of Food Sciences, 38(3), 171–178. 10.17221/269/2019-CJFS

[fsn34095-bib-0005] Adhikari, B. , Howes, T. , Bhandari, B. R. , & Truong, V. (2001). Stickiness in foods: A review of mechanisms and test methods. International Journal of Food Properties, 4(1), 1–33. 10.1081/JFP-100002186

[fsn34095-bib-0006] Akhtar, N. , Adnan, Q. , Ahmod, M. , Mahmood, A. , & Farsana, K. (2009). Rheological studies and characteristics of different oils. Journal of the Chemical Society of Pakistan, 31(2), 201–206.

[fsn34095-bib-0007] Aly, M. M. A. , & Seleem, H. A. (2015). Gluten‐free flat bread and biscuits production by cassava, extruded soy protein and pumpkin powder. Food and Nutrition Sciences, 6(7), 660–674. 10.4236/fns.2015.67069

[fsn34095-bib-0008] Amagloh, F. K. , Mutukumira, A. N. , Brough, L. , Weber, J. L. , Hardacre, A. , & Coad, J. (2013). Carbohydrate composition, viscosity, solubility and sensory acceptance of sweet potato‐ and maize‐based complementary foods. Food & Nutrition Research, 57, 1–10. 10.3402/fnr.v57i0.18717 PMC3600427 23516115

[fsn34095-bib-0009] Amevor, P. M. , Laryea, D. , & Barimah, J. (2018). Sensory evaluation, nutrient composition and microbial load of cashew nut–chocolate spread. Cogent Food & Agriculture, 4, 1480180–1480190. 10.1080/23311932.2018.1480180

[fsn34095-bib-0010] AOAC . (2010). Official methods of analysis of the Association of Official Analytical Chemists (18th ed.). Association of Official Analytical Chemists.

[fsn34095-bib-0011] Aydin, S. , & Özdemir, Y. (2017). Development and characterization of carob flour based functional spread for increasing use as nutritious snack for children. Journal of Food Quality, 2017, 1–7. 10.1155/2017/5028150

[fsn34095-bib-0012] Babarykin, D. , Smirnova, G. , Pundinsh, I. , Vasiljeva, S. , Krumina, G. , & Agejchenko, V. (2019). Red beet (*Beta vulgaris*) impact on human health. Journal of Biosciences and Medicines, 7, 61–79.

[fsn34095-bib-0013] Bascuas, S. , Espert, M. , Llorca, E. , Quiles, A. , Salvador, A. , & Hernando, I. (2021). Structural and sensory studies on chocolate spreads with hydrocolloid‐based oleogels as a fat alternative. LWT ‐ Food Science and Technology, 135, 110228–110235.

[fsn34095-bib-0014] Bourne, M. C. (2003). Food texture and viscosity: Concept and measurement. Elsevier Press.

[fsn34095-bib-0015] Caliskan, A. , Abdullah, N. , & Ishak, N. (2022). Physicochemical properties of cypriot wild carob (*Ceratonia siliqua* L.) powder as cocoa powder substitute. International Journal of Latest Research in Humanities and Social Science (IJLRHSS), 5(6), 145–154. https://www.researchgate.net/publication/361856247

[fsn34095-bib-0016] Chaiyasit, W. , Elias, R. J. , McClements, D. J. , & Decker, E. A. (2007). Role of physical structures in bulk oils on lipid oxidation. Critical Reviews in Food Science and Nutrition, 47(3), 299–317. 10.1080/10408390600754248 17453926

[fsn34095-bib-0017] CODEX STAN 87‐1981 . (2003). Codex standards for cocoa products and chocolate. World Health Organization. p. 7.

[fsn34095-bib-0018] Cozentino, I. S. C. , Paula, A. V. , Ribeiro, C. A. , Alonso, J. D. , Grimaldi, R. , Luccas, V. , Taranto, M. P. , & Cavallini, D. C. U. (2022). Development of a potentially functional chocolate spread containing probiotics and structured triglycerides. LWT ‐ Food Science and Technology, 154, 112746–112754. 10.1016/j.lwt.2021.112746

[fsn34095-bib-0019] Dubost, N. J. , Shewelt, R. L. , & Eitenmiller, R. R. (2003). Consumer acceptability, sensory and instrumental analysis of peanut soy spreads. Journal of Food Quality, 26, 27–42. 10.1111/j.1745-4557.2003.tb00224.x

[fsn34095-bib-0020] Eagan, H. R. S. , & Kir, S. R. (1981). Pearson's chemical analysis of foods. Church Hill Livingstone Pub.

[fsn34095-bib-0021] Eghdami, A. , & Sadeghi, F. (2010). Determination of total phenolic and flavonoids contents in methanolic and aqueous extract of *Achillea millefolium* . Journal of Organic Chemistry, 2, 81–84.

[fsn34095-bib-0022] El‐Mesallamy, A. M. D. , Abd El‐Latif, A. E. , Abd ElAzim, M. H. , Mahdi, M. G. M. , & Hussein, S. A. M. (2020). Chemical composition and biological activities of red beetroot (*Beta vulgaris* Linnaeus) roots. Egyptian Journal of Chemistry, 63(1), 239–246. 10.21608/ejchem.2019.17977.2092

[fsn34095-bib-0023] Fischer, S. , Wilckensa, R. , Jara, J. , & Arandac, M. (2013). Variation in antioxidant capacity of quinoa (*Chenopodium quinoa* Will) subjected to drought stress. Industrial Crops and Products, 46, 341–349. 10.1016/j.indcrop.2013.01.037

[fsn34095-bib-0024] García‐Díez, E. , Sánchez‐Ayora, H. , Blanch, M. , Ramos, S. , & Martín, M. Á. (2022). Exploring a cocoa–carob blend as a functional food with decreased bitterness: Characterization and sensory analysis. LWT ‐ Food Science and Technology, 165, 113708–113716.

[fsn34095-bib-0025] Geller, D. P. , & Goodrum, J. W. (2000). Rheology of vegetable oil analogs and triglycerides. Journal of the American Oil Chemists' Society, 77(2), 111–114.

[fsn34095-bib-0026] Glibowski, P. , Zarzycki, P. , & Krzepkowska, M. (2008). The rheological and instrumental textural properties of selected table fats. International Journal of Food Properties, 11(3), 678–686. 10.1080/10942910701622599

[fsn34095-bib-0027] Gul, B. , Khan, S. , & Ahmad, I. (2022). Extraction of phytochemicals from date palm (*Phoenix dactylifera* L.) seeds by enzymatic hydrolysis. Journal of Food Processing and Preservation, 46(11), 1–9. 10.1111/jfpp.17007

[fsn34095-bib-0028] Gulzar, N. , Sameen, A. , Khan, M. I. , Huma, N. , Murtaza, M. , & Rafiq, S. (2015). Nutritional and functional properties of fruited cream cheese spread as influenced by hydrocolloids. Journal of Food and Nutrition Research, 3(3), 191–195.

[fsn34095-bib-0029] Hamid, A. , & Damit, A. A. (2004). Quality of Malaysian cocoa butter during storage. Journal of the Science of Food and Agriculture, 84, 513–516. 10.1002/jsfa.1629

[fsn34095-bib-0030] Higazy, M. M. E. , EL. Diffrawy, A. A. M. , Zeitoun, M. A. M. , Shaltout, O. E. , & Abou El‐Yazeed, A. M. (2018). Nutrients of carob and seed powders and its application in some food products. Journal of the Advances in Agricultural Researches (Faculty of Agriculture Saba Basha), 23(1), 130–147.

[fsn34095-bib-0031] Ibrahim, O. S. , Mohammed, A. T. , & Abd‐Elsattar, H. H. (2015). Quality characteristics of rice biscuits sweetened with carob powder. Middle East Journal of Applied Sciences, 5(4), 1082–1090.

[fsn34095-bib-0032] Ibrahim, R. M. (2022). Utilization of chickpea split (*Cicer arietinum* L.) in preparing some gluten‐free casein‐free food products for autism children. Food and Nutrition Sciences, 13, 284–315. 10.4236/fns.2022.133023

[fsn34095-bib-0033] Ibrahim, R. M. , Abdel‐Salam, F. F. , & Farahat, E. (2020). Utilization of carob (*Ceratonia siliqua* L.) extract as functional ingredient in some confectionery products. Food and Nutrition Sciences, 11, 757–772. 10.4236/fns.2020.118054

[fsn34095-bib-0034] Issaoui, M. , Flamini, G. , & Delgado, A. (2021). Sustainability opportunities for mediterranean food products through new formulations based on carob flour (*Ceratonia siliqua* L.). Sustainability, 13(14), 8026–8047. 10.3390/su13148026

[fsn34095-bib-0035] Jahan, T. A. , Vandenberg, A. , Glahn, R. P. , Tyler, R. T. , Reaney, M. J. T. , & Tar'an, B. (2019). Iron fortification and bioavailability of chickpea (*Cicer arietinum* L.) seeds and flour. Nutrients, 11(9), 2240–2257. 10.3390/nu11092240 31540391 PMC6770251

[fsn34095-bib-0036] James, C. S. (1995). General food studies. In Analytical chemistry of foods, Blachie academic and professiona, London, New York, Tokyo, Chapter 6, p. 135.

[fsn34095-bib-0037] Kale, R. G. , Sawate, A. R. , Kshirsagar, R. B. , Patil, B. M. , & Mane, R. P. (2018). Studies on evaluation of physical and chemical composition of beetroot (*Beta vulgaris* L.). International Journal of Chemical Studies, 6(2), 2977–2979. https://www.researchgate.net/publication/325057965

[fsn34095-bib-0038] Kaur, R. , & Prasad, K. (2021). Nutritional characteristics and value‐added products of chickpea (*Cicer arietinum*)—A review. Journal of Postharvest Technology, 9(2), 1–13.

[fsn34095-bib-0039] Khalil, R. A. M. , & Elkot, W. F. (2020). Functional properties and nutritional quality of processed cheese spreads enriched with black rice powder. Egyptian Journal of Food Science, 48(2), 281–289. 10.21608/ejfs.2020.36261.1068

[fsn34095-bib-0040] McGurie, R. G. (1992). Reporting of objective color measurements. HortScience, 27, 1254–1255. 10.21273/HORTSCI.27.12.1254

[fsn34095-bib-0041] Mohidem, N. A. , Hashim, N. , Shamsudin, R. , & Man, H. C. (2022). Rice for food security: Revisiting its production, diversity, Rice milling process and nutrient content. Agriculture, 12, 741–768. 10.3390/agriculture12060741

[fsn34095-bib-0042] Morais, A. C. S. , & Rodrigues, M. C. P. (2018). Optimization and consumer acceptability of carob powder as cocoa substitute in lactose‐free cashew nut almonds‐based beverage. International Food Research Journal, 25(6), 2268–2274.

[fsn34095-bib-0043] Murugkar, D. A. (2014). Effect of sprouting of soybean on the chemical composition and quality of soymilk and tofu. Journal of Food Science and Technology, 51(5), 915–921. 10.1007/s13197-011-0576-9 24803698 PMC4008751

[fsn34095-bib-0044] Neha, P. , Jain, S. K. , Jain, N. K. , Jain, H. K. , & Mittal, H. K. (2018). Chemical and functional properties of beetroot (*Beta vulgaris* L.) for product development: A review. International Journal of Chemical Studies, 6(3), 3190–3194.

[fsn34095-bib-0045] Nsimba, R. Y. , Kikuzaki, H. , & Konishi, Y. (2008). Antioxidant activity of various extracts fractions of *Chenopodium quinoa* and *Amaranthus* spp. seeds. Food Chemistry, 106, 760–766. 10.1016/j.foodchem.2007.06.004

[fsn34095-bib-0046] Olusola, O. (2007). Effect of refining on the physical and chemical properties of cashewkernel oil. International Journal of Food Science & Technology, 16, 513–517. 10.1111/j.1365-2621.1981.tb01844.x

[fsn34095-bib-0047] Panda, D. K. , Jyotirmayee, B. , & Mahalik, G. (2022). Black rice: A review from its history to chemical makeup to health advantages, nutritional properties and dietary uses. Plant Science Today, 9, 1–15. 10.14719/pst.1817

[fsn34095-bib-0048] Pandey, G. S. , Patil, M. T. , Vir, D. K. , Pandey, P. M. , & Pathan, R. A. (2020). Exploring formulation and evaluation of simethicone medicated chocolate formulation for antiflatulence effect. World Journal of Pharmaceutical Research, 9(14), 970–985. 10.20959/wjpr202014-19146

[fsn34095-bib-0049] Radfar, R. , Farhoodi, M. , Ghasemi, I. , Khaneghah, A. M. , Shahraz, F. , & Hosseini, H. (2019). Assessment of phenolic contents and antioxidant and antibacterial activities of extracts from four varieties of iranian date palm (*Phoenix dactylifera* L.) seeds. Applied Food Biotechnology, 6(3), 173–184. 10.22037/afb.v6i3.23379

[fsn34095-bib-0050] Redgwell, R. J. , & Hansen, C. E. (2000). Isolation and characterisation of cell wall polysaccharides from cocoa (*Theobroma cacao* L.) beans. Planta, 210(5), 823–830. 10.1007/s004250050685 10805455

[fsn34095-bib-0051] Şahin, S. , & Özata, A. B. (2022). Substitution of cocoa powder with hazelnut skin powder in cocoa hazelnut spreads. Journal of Food Processing and Preservation, 46(12), e17276–e17287. 10.1111/jfpp.17276

[fsn34095-bib-0052] Said, A. , Nasir, N. A. M. , Abu Bakar, C. A. , & Mohamad, W. A. F. W. (2019). Chocolate spread emulsion: Effects of varying oil types on physico‐chemical properties, sensory qualities and storage stability. Journal of Agrobiotechnology, 10(2), 32–42.

[fsn34095-bib-0053] Shehata, M. M. E. M. (2020). Influence of fortification of biscuits with *Hyphaene thebaica* flour on quality attributes biochemical parameters and histological examination of pancreas in diabetic rats. Journal of Home Economics, 36(1), 47–70. 10.21608/jhe.2020.116419

[fsn34095-bib-0054] Singh, R. P. , Murthy, K. N. C. , & Jayaprakasha, G. K. (2002). Studies on antioxidant activity of pomegranate (*Punica granatum*) peel and seed extracts using in vitro models. Journal of Agricultural and Food Chemistry, 50, 81–86. 10.1021/jf010865b 11754547

[fsn34095-bib-0055] Tarakçi, Z. , & Yildirim, M. (2020). Efects of ghee and olive oil usage on cocoa hazelnut spreads during storage. Journal of Food Processing and Preservation, 45, e15246–e15256. 10.1111/jfpp.15246

[fsn34095-bib-0056] Tizazu, S. , Urga, K. , Abuye, C. , & Retta, N. (2010). Improvement of energy and nutrient density of sorghum based complementary food using germination. African Journal of Food Agriculture Nutrition and Development, 10(8), 2927–2942. 10.4314/ajfand.v10i8.60875

[fsn34095-bib-0057] Walker, R. , Tseng, A. , Cavender, G. , Ross, A. , & Zhao, Y. (2014). Physicochemical, nutritional and sensory qualities of wine grape pomace fortified baked goods. Journal of Food Science, 79(9), S1811–S1822. 10.1111/1750-3841.12554 25102950

[fsn34095-bib-0058] Yu, S. , Xu, J. , Zhang, Y. , & Kopparapu, N. K. (2014). Relationship between intrinsic viscosity, thermal and retrogradation properties of amylose and amylopectin. Czech Journal of Food Sciences, 32, 514–520. 10.17221/394/2013-CJFS

[fsn34095-bib-0059] Zoani, C. , Anorga, L. , Belc, N. , Castanheira, I. , Donard, O. F. X. , Kourimska, L. , Kukovecz, A. , Iatco, I. , Najdenkoska, A. , Ogrinc, N. , Ozer, H. , Rychlik, M. , Tsimidou, M. Z. , Van Loco, J. , & Zappa, G. (2018). Feasibility studies for new food matrix‐reference materials. Journal of Physics: Conference Series, 1065, 232005. 10.1088/1742-6596/1065/23/232005

[fsn34095-bib-0060] Zyzelewicz, D. , Nebesny, E. , Motyl, I. , & Libudzisz, Z. (2010). Effect of milk chocolate supplementation with lyophilised lactobacillus cells on its attributes. Czech Journal of Food Sciences, 28(5), 392–406. 10.17221/217/2009-CJFS

